# Effects of Shoes That Can Be Tightened Using Wire and Dial on the Dynamic Balance Following Ankle Muscle Fatigue: A Crossover Study

**DOI:** 10.3390/healthcare9050578

**Published:** 2021-05-13

**Authors:** Im-Rak Choi, Jung-Hoon Lee

**Affiliations:** 1 Department of Rehabilitation Therapy Team, Sports Exercise Therapy Center, Good Samsun Hospital, Busan 47007, Korea; irchoi@hanmail.net; 2 Department of Physical Therapy, College of Nursing, Healthcare Science and Human Ecology, Dong-Eui University, Busan 47340, Korea

**Keywords:** dynamic balance, ankle, wire, shoes, muscle fatigue

## Abstract

Ankle muscle fatigue causes joint instability and increased postural sway, which triggers imbalance, leading to injury. The purpose of this study was to investigate the immediate effects of wearing shoes that can be tightened using wire and dial (SWD) compared to being barefoot and wearing lace shoes of the slip-on type (LSS) on the dynamic balance of the ankle after muscle fatigue. Twenty-two healthy individuals were enrolled in this study. Muscle fatigue in the ankle was induced using Biodex, an isokinetic equipment. The participants were randomly allocated to the barefoot, LSS, and SWD groups, and the dynamic balance immediately after inducing muscle fatigue in each participant was measured using BIORescue, the Y-Balance test, and the side-hop test. The results showed that after inducing ankle muscle fatigue, wearing SWD leads to a more significant increase in dynamic balance than barefoot and wearing LSS (*p* < 0.05). Hence, to improve the dynamic balance of the ankle after muscle fatigue, wearing SWD is suggested as it allows the tightening of the ankle and dorsum of the foot using the wire and dial.

## 1. Introduction

Activities of daily living and sports activities are limited by injuries to the ankle, which is prone to frequent injuries during such activities [1]. The ankle is also critical for postural control, while the ankle muscles maintain balance in a standing posture via small movements [2,3,4]. Balance is the ability to maintain the vertical center of mass and perform movements within the support base [5]. The muscles involved in dorsiflexion and plantarflexion play a particularly important role in maintaining balance [4].

Muscle fatigue is caused by persistent and repetitive movements, which reduce the maximum strength and performance of the muscle [6,7,8]. Moreover, it increases the risk of pain and injury [9,10], with potential damage induced by proprioceptive and neurological changes [11]. Muscle fatigue leads to reduced stability and maintenance of posture during the movement as well as a muted response to external stimuli [12]. The compromised neuromusculoskeletal regulation thus impacts the balance [13]. When muscle fatigue is induced on the ankle joint, the consequent joint instability and increased postural sway reduce the ability to maintain the balance [14], while the increased sway can cause injuries [15,16].

Various methods have been used to improve the stability of the ankle, including stretching [17], exercise [18], taping [19], and joint mobilization [20]. A recent study investigated the effects of Ankle Foot Orthosis (AFO), wherein the ankle could be tightened using a wire and dial in patients with stroke, and showed that the patients using AFO who had wire and dial showed better improvement in balance control compared to the patients using the conventional AFO [21]. AFO is uncomfortable when worn due to its plastic material and large volume [22], and it rather decreases the dynamic balance [23]. While many shoes with the wire and dial for tightening the ankle and dorsum of the foot are available, no study has investigated the effects of such shoes on maintaining balance. Thus, the present study aimed to compare the immediate effects of being barefoot, wearing elastic lace shoes of the slip-on type (LSS) that can be worn and taken off with ease, but lack stability, and wearing elastic shoes that can be tightened using a wire and dial (SWD) to provide stability to the ankle for dynamic balance in healthy male adults in whom ankle muscle fatigue of the dominant leg was induced using an isokinetic equipment.

## 2. Materials and Methods

### 2.1. Participants

The sample size in this study was estimated using the G*Power 3.1 program based on Cohen’s power analysis [24]. The estimated sample size was 21 with an effect size of 0.8, significance level 0.05, and power 90%. Considering the probable dropout rate, a total of 25 participants were recruited [24].

Healthy male adults aged ≥20 years were selected based on the following. Inclusion criteria: (1) no back, knee, or ankle pain, (2) normal nervous and musculoskeletal systems, (3) no history of drug intake within the past year, and (4) no deformity in the lower extremities. Three participants were excluded after recruitment as they did not satisfy the inclusion criteria. This study was conducted with the approval of the Dongeui University (Busan, Korea) Institutional Review Board (DIRB-20208_HR-R-39).

### 2.2. Study Design

**Figure 1:**
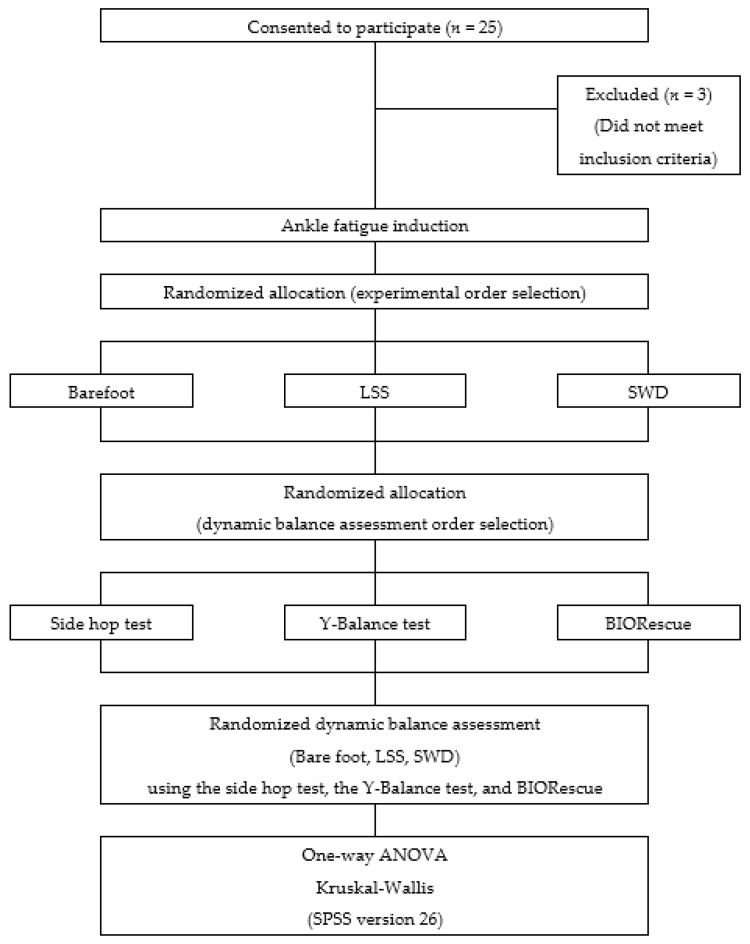
Flow chart of study. LSS: lace shoes of the slip-on type; SWD: shoes that can be tightened using wire and dial; ANOVA: analysis of variance. SPSS (IBM Corp., Armonk, NY, USA).

To investigate the immediate effects of being barefoot, and wearing LSS and SWD on dynamic balance, a crossover study design was followed. First, muscle fatigue was induced on the ankle joint of the participants’ dominant leg using Biodex (Biodex system 4, Biodex Medical System Inc., New York, NY, USA), an isokinetic equipment. The participants were randomly allocated to the barefoot, LSS, or SWD groups on the basis of the computer-generated random numbers. The side-hop test, the Y-Balance test, and BIORescue (RM Ingeniere, Rodes, France) were used to assess the dynamic balance, and the test order was also selected on the basis of the computer-generated random numbers. Between each measurement, the participants were allowed a minute’s rest. The experimental design and procedures are presented as a flow chart in Figure 1.

### 2.3. Shoes

#### 2.3.1. Shoes That Can Be Tightened Using Wire and Dial (SWD)

**Figure 2:**
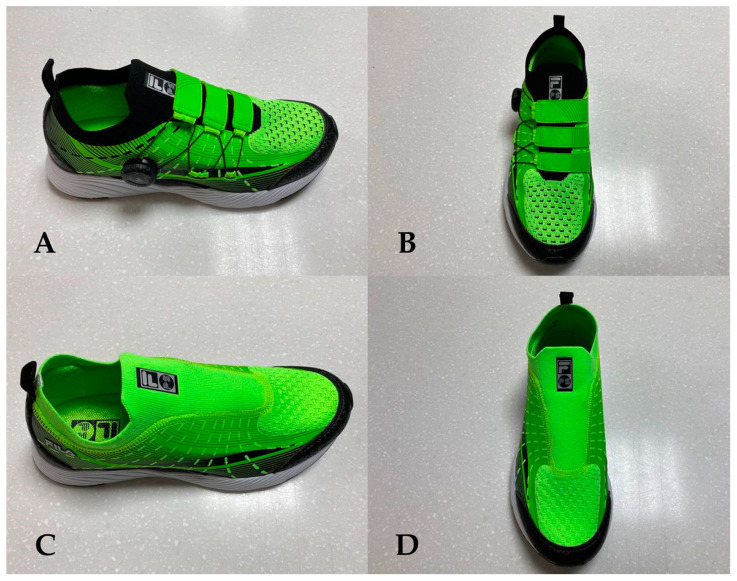
(**A**): Lateral view of shoes that can be tightened using a wire and dial; (**B**): anterior view of shoes that can be tightened using a wire and dial; (**C**): lateral view of lace shoes of the slip-on type; (**D**): anterior view of lace shoes of the slip-on type.

SWD are made of knit material with excellent elasticity with the energized midsole providing comfort and a cushioning effect (FILA, Korea Co., Ltd., Seoul, Korea). The Velcro-shaped straps on the front are connected through a wire, and a dial is used to tighten the wire to stabilize the ankle and dorsum of the foot, which is known as the FREELOCK fit system (Shinkyung Inc., Busan, Korea) (Figure 2A,B).

#### 2.3.2. Lace Shoes of the Slip-on Type (LSS) Based on Elastic Material

LSS are made of a knit material with excellent elasticity (FILA, Korea Co., Ltd., Seoul, Korea) that can be worn and taken off with ease and provide excellent comfort. The energized midsole provides extra comfort and a cushioning effect (Figure 2C,D).

### 2.4. Measurement

#### 2.4.1. Muscle Fatigue

**Figure 3:**
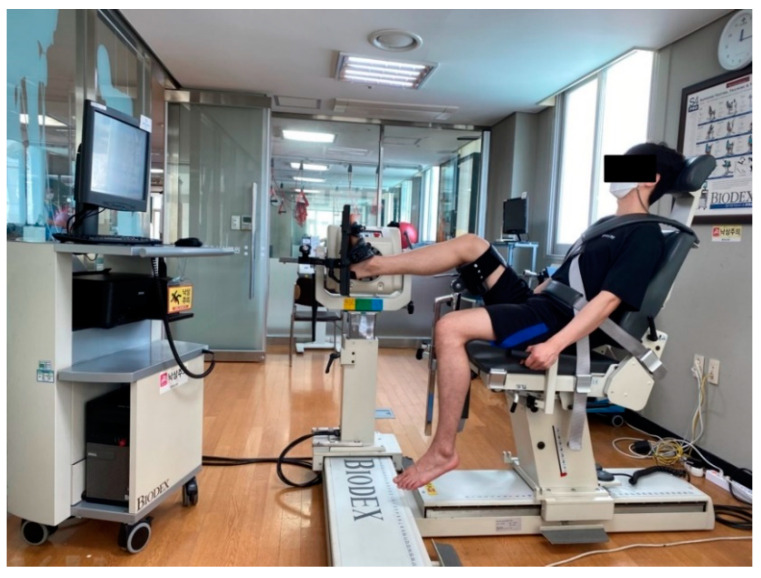
Ankle muscle fatigue induced using Biodex.

Muscle fatigue was induced on the ankle of the dominant leg using an isokinetic equipment Biodex (Figure 3). Biodex has been reported to show high test-retest reliability (ICC = 0.82~0.95) [25], while it determines the peak torque by measuring the highest value against the resistance to movement with constant speed within the set joint range of motion. Maximum peak torque was measured at 60°/s before inducing the ankle muscle fatigue; subsequently, ankle exercise at 60°/s was performed repeatedly. Since each individual has a different number of Biodex repetitions that cause muscle fatigue, muscle fatigue was defined as the peak torque reaching half the maximum level [26].

#### 2.4.2. BIORescue

**Figure 4:**
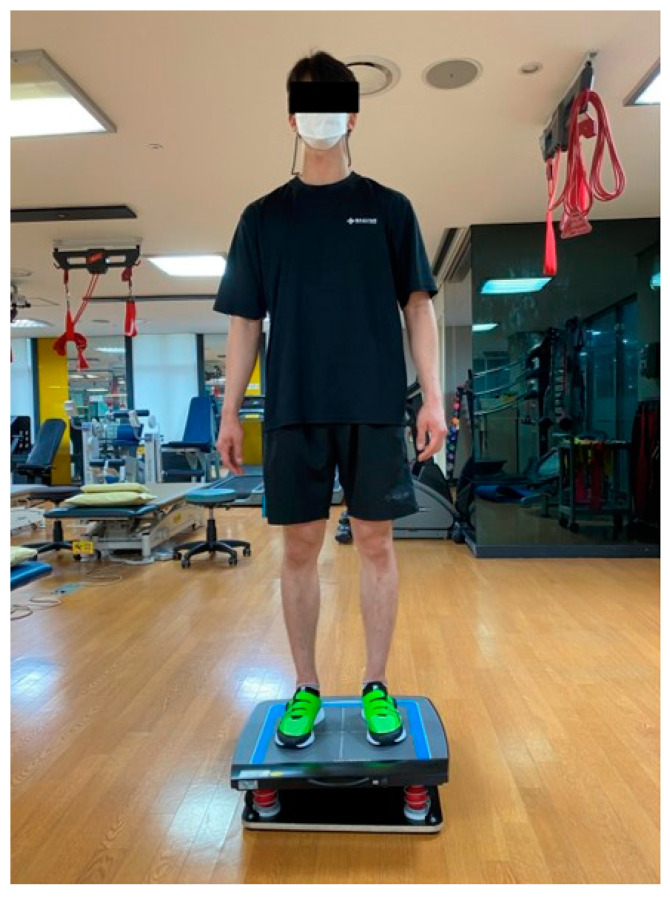
Ankle dynamic balance assessment using BIORescue.

BIORescue is a device that measures the balance of human body by sensing the change in the center of pressure through a plate (610 mm × 580 mm × 10 mm) with 1600 sensors. Each sensor operated independently and the pressure measurement range was 1–100 N/cm^2^. A lower surface area ellipse (mm^2^) indicates a better balance [27,28]. The participant was instructed to stand on a plate located 1 m away from the computer while looking straight ahead to measure the surface area ellipse. The measurement is taken for 30 s (Figure 4). The device reliability is ICC = 0.83~0.95 [29].

#### 2.4.3. Y-Balance Test

**Figure 5:**
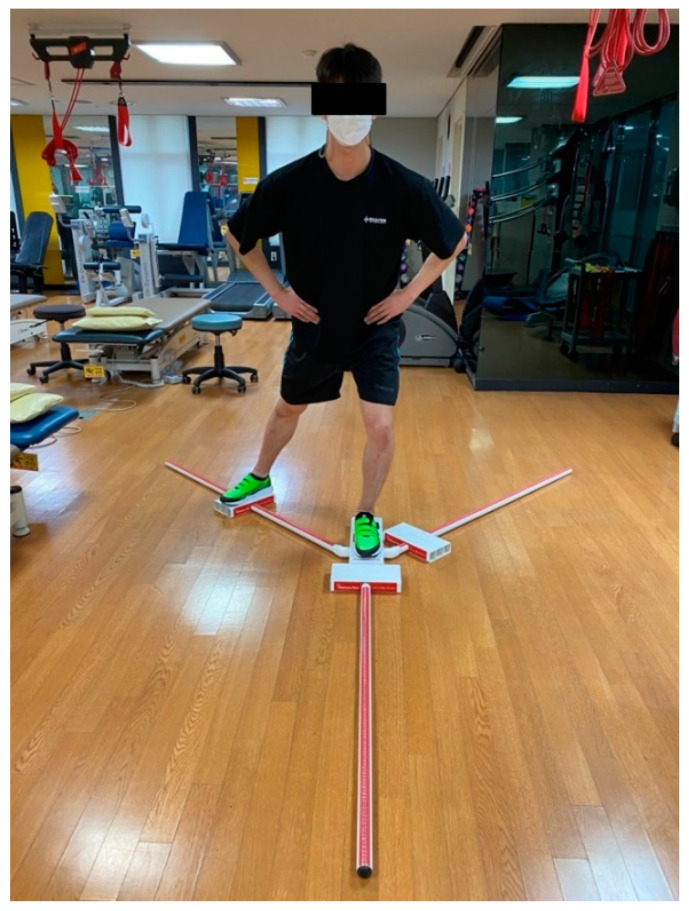
Ankle dynamic balance assessment using the Y-Balance kit.

Y-Balance test is commonly used to measure the strength, flexibility, and proprioception of the lower extremity. It is a modified version of the star excursion balance test (SEBT) with better repeatability [30]. Among the eight directions of the SEBT, the anterior, posteromedial, and posterolateral directions, which show high reliability in assessing chronic ankle instability (ICC = 0.91), were used [31,32]. The stance platform of the Y-Balance Test Kit (Move2Perform, Evansville, IN, USA) has three PVC pipes connected in anterior, posteromedial, and posterolateral directions, while the anterior pipe is at an angle of 135° with the posterior pipes. Before inducing muscle fatigue, the participant was guided to practice the Y-Balance in each direction three times, following which a random direction was selected, and the distance of the leg stretching by the participant was measured. The fatigue-inducing ankle of the dominant leg was supported by the Y-Balance Test Kit, and the stretching distance of the other leg was measured. If the supporting leg did not maintain contact with the ground or the stretched leg touched the ground for regaining balance, or if the stretched leg did not return to the original position, the measurement was considered as null and was repeated [30] (Figure 5). After inducing muscle fatigue on the ankle of the dominant leg, the same method was applied for the measurements in each direction of the Y-Balance test. To standardize the result values for the differences in individual leg length, the following formula was used: {(anterior + posteromedial + posterolateral)/leg length} × 100 [33].

#### 2.4.4. Side-Hop Test

**Figure 6:**
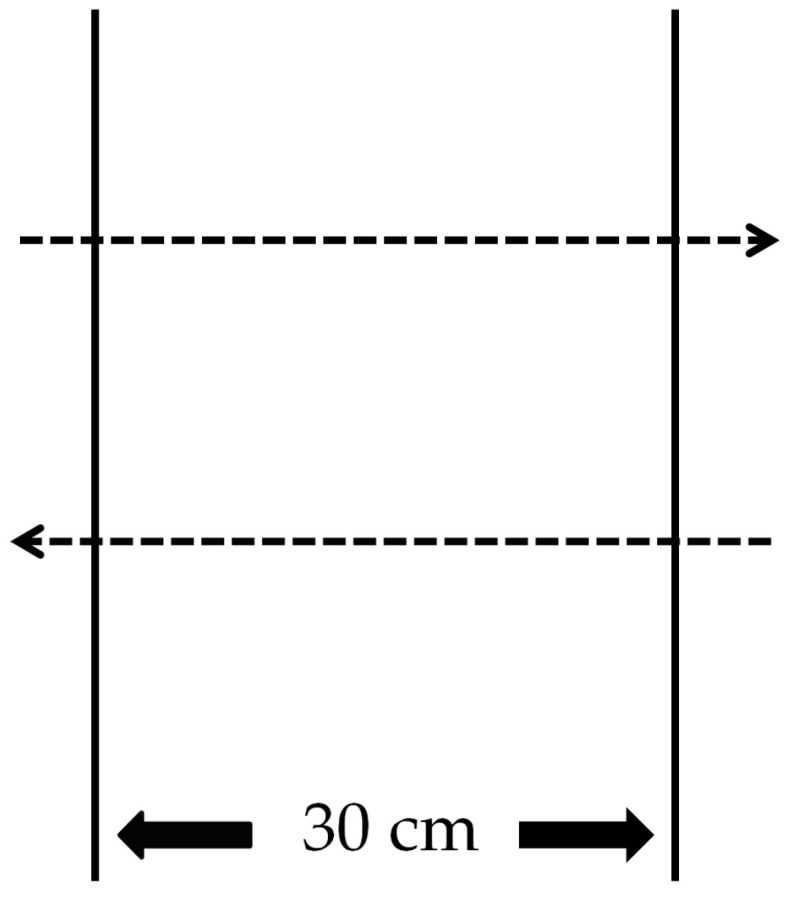
Ankle dynamic balance assessment using the side-hop test.

A side-hop test is performed to assess the inversion-eversion that occurs on the frontal and transverse planes. In this test, a hop of 30 cm distance on one leg to return to the original position was considered as one set, and the time to repeat 10 sets of hops was measured (ICC = 0.84) [34]. Starting immediately next to the line, the faster the time to repeat 10 times, the better the dynamic balance [34]. For the minimum learning effect, the participant was guided to practice the side hop three times before inducing muscle fatigue. If the line was stepped on, the measurement was considered null and was repeated after a rest of 30 s [34] (Figure 6).

### 2.5. Statistical Analysis

For data processing and analysis, the SPSS 26.0 (Version 26.0 for Windows, IBM Corp., Armonk, NY, USA) was used, with the level of significance set to 0.05. Descriptive statistics were used for the participants’ general characteristics.

The data obtained from the Y-Balance test showed normal distribution in the Shapiro–Wilk normality test at *p* > 0.05. To analyze the effects of being barefoot, and wearing LSS and SWD on dynamic balance, one-way analysis of variance was used, and Tukey’s test was used as the post hoc test for multiple comparisons.

The data obtained from BIORescue and the side-hop test did not show normal distribution in the Shapiro–Wilk normality test (*p* < 0.05). Thus, the Kruskal–Wallis test was used to analyze the effects of being barefoot, and wearing LSS and SWD on dynamic balance, and the Mann–Whitney test was used as the post hoc test.

## 3. Results

### 3.1. General Characteristics

The average age of the 22 male participants was 26.77 ± 3.21 years. The mean height and weight were 176.32 ± 5.21 cm and 74.5 ± 9.26 kg, respectively. The dominant leg was the right leg for 18 participants (81.81%) and the left leg for 4 participants (18.19%), with a mean foot size of 268.64 ± 6.76 mm (Table 1).

### 3.2. Dynamic Balance Using Y-Balance

The dynamic balance measured using the Y-Balance test for being barefoot, and wearing LSS and SWD, showed a significant difference between the anterior, posteromedial, and posterolateral directions (*p* < 0.05). The post hoc test found a significant difference between being barefoot and wearing SWD (*p* = 0.000) and between wearing LSS and SWD (*p* = 0.013) but not between being barefoot and wearing LSS (*p* = 0.133) for the anterior direction. For the posteromedial direction, a significant difference was found between being barefoot and wearing SWD (*p* = 0.000) and between wearing LSS and SWD (*p* = 0.009) but not between being barefoot and wearing LSS (*p* = 0.154). For the posterolateral direction, a significant difference was found only between being barefoot and wearing SWD (*p* = 0.001) and not between wearing LSS and SWD (*p* = 0.053) or between being barefoot and wearing LSS (*p* = 0.281) (Table 2).

The leg length reach % showed a significant difference, and the post hoc test found a significant difference between being barefoot and wearing SWD (*p* = 0.000) and between wearing LSS and SWD (*p* = 0.044), but not between being barefoot and wearing LSS (*p* = 0.119) (Table 2).

### 3.3. Dynamic Balance Using Side-Hop Test

The dynamic balance measured using the side-hop test showed a significant difference (*p* < 0.05). The post hoc test found a significant difference between being barefoot and wearing SWD (*p* = 0.000) and between wearing LSS and SWD (*p* = 0.012), but not between being barefoot and wearing LSS (*p* = 0.028) (Table 3).

### 3.4. Dynamic Balance Using BIORescue

The dynamic balance measured using BIORescue showed a significant difference (*p* < 0.05). The post hoc test found a significant difference between being barefoot and wearing LSS (*p* = 0.005), between wearing LSS and SWD (*p* = 0.006), and between being barefoot and wearing SWD (*p* = 0.000) (Table 4).

## 4. Discussion

In this study, wearing SWD after muscle fatigue on the ankle of the dominant leg showed an immediate improvement in dynamic balance to a greater level than barefoot or wearing LSS. The ankle supports the weight of the body, absorbs shocks and maintains balance when during movement, when the foot touches the ground [35]. However, when muscle fatigue occurs in the ankle, the acuity of the ankle position sense decreases [36], and adverse effects on postural control occur by reducing the force sense [37], destabilizing the ankle joint and increasing medial–lateral postural fluctuations, resulting in a decrease in the balance ability [14]. As in this study, after the development of isokinetic fatigue of the dorsiflexors and plantarflexors in healthy young men, there was a negative effect on the postural sway in terms of the range of postural control and balance maintenance [14]. Muscle fatigue in plantarflexors is said to increase postural sway by interfering with the stability of the ankle joint [38]. In a previous study, in order to provide mechanical stability of the ankle after inducing muscle fatigue in the ankle of normal adults, the balance ability was increased by an intervention to control the inversion and eversion of the ankle [19], although this study was not applied to healthy adults with muscle fatigue at the ankle; in a previous study the use of AFO using a wire and dial, rather than the conventional plastic AFO, showed an improvement in the static balance measured using the BIORescue in stroke patients with foot drop [21]. The stability of the joint increased as the AFO wire on the medial and lateral sides of the ankle was controlled using the dial to adjust the position of the ankle joint, while covering the entire foot to press or fix the foot joint [21].

The SWD used in this study has a Velcro-shaped strap connected to the shoe string through a wire so that the use of the dial to tighten the wire can press and fix the area from the dorsum of the foot to the ankle. The increase in the dynamic balance might be attributed to the increased stability of the medial and lateral sides of the ankle and the subtalar joint with a simultaneous increase in ankle joint stability, as the inversion and eversion of the ankle after muscle fatigue were adjusted by a decrease in the overall volume of shoes with a tightened wire.

The limitations of this study are as follows. First, the results cannot be generalized as the study investigated the muscle fatigue induced on the ankle of the dominant leg only in healthy male adult participants. Second, only the immediate improvement in dynamic balance was measured after inducing muscle fatigue on the ankle, and whether the effect was maintained could not be verified. Third, because the level of tightening of the wire using the dial to improve the dynamic balance could not be accurately quantified, a subsequent study on the application intensity of the tightening is necessary. Fourth, muscle fatigue was induced only in ankle, and a balance test was performed using the ankle, knee and hip joints and the compensatory action of the knee and hip joints could not be controlled. Fifth, there are no previous studies on shoes that can be tightened using wires and dials; therefore, there are no comparable data. Sixth, we studied only 22 healthy men; further studies with a larger number of subjects and patients are necessary.

## 5. Conclusions

The dynamic balance after induced ankle muscle fatigue of the dominant leg showed an immediate improvement with wearing SWD to a greater extent than with barefoot or wearing LSS. However, further studies regarding the wearing of SWD are necessary, as it allows the tightening of the ankle and dorsum of the foot using a wire and dial to improve the dynamic balance following ankle muscle fatigue.

## Figures and Tables

**Table 1:** General characteristics of participants (*n* = 22).

Variables	Mean ± SD or Mode (%)
Male	22 (100)
Female	0 (0)
Age (years)	26.77 ± 3.21
Height (cm)	176.32 ± 5.21
Weight (kg)	74.5 ± 9.26
Dominant leg (Right)	18 (81.81)
Dominant leg (Left)	4 (18.19)
Foot size (mm)	268.64 ± 6.76

SD: standard deviation.

**Table 2:** Dynamic balance of the dominant leg using the Y-Balance test.

	Bare Foot	LSS	SWD	*p*-Value
Anterior direction (cm)	61.07 ± 7.56	65.29 ± 7.02	71.66 ± 8.32	0.000
Posteromedial direction (cm)	93.41 ± 8.39	98.18 ± 7.79	105.98 ± 9.06	0.000
Posterolateral direction (cm)	101.02 ± 10.13	105.39 ± 8.91	112.14 ± 9.17	0.001
Leg length reach %	296.32 ± 36.34	316.06 ± 29.34	340.17 ± 36.82	0.000

Values are presented as mean ± standard deviation. LSS: lace shoes of the slip-on type; SWD: shoes that can be tightened using wire and dial.

**Table 3:** Dynamic balance using side-hop test.

	Bare Foot	LSS	SWD	*p*-Value
Side-hop test(s)	16.19 ± 4.68	13.96 ± 4.29	11.57 ± 3.26	0.000

Values area presented as mean ± standard deviation. LSS: lace shoes of the slip-on type; SWD: shoes that can be tightened using wire and dial.

**Table 4:** Dynamic balance using BIORescue.

	Bare Foot	LSS	SWD	*p*-Value
Surface area ellipse (mm^2^)	371.86 ± 199.67	212.82 ± 98.74	138.68 ± 86.27	0.000

Values area presented as mean ± standard deviation. LSS: lace shoes of slip-on type; SWD: shoes that can be tightened using wire and dial.

## Data Availability

The data presented in this study are available in insert article.
